# Assessment of job satisfaction among pharmacy professionals

**DOI:** 10.1186/s40545-021-00356-1

**Published:** 2021-08-21

**Authors:** Muluwork Sahile Berassa, Tebeje Ashegu Chiro, Selamawit Fanta

**Affiliations:** 1 Department of Pharmaceutics and Social Pharmacy, School of Pharmacy, College of Health Sciences, Addis Ababa University, Addis Ababa, Ethiopia; 2 School of Nursing and Midwifery, College of Medicine and Health Sciences, Hawassa University, Hawassa, Ethiopia; 3 Alamata General Hospital, Raya Azebo Zone, Mekelle, Tigray Region Ethiopia

**Keywords:** Pharmacy professionals, Job satisfaction, Tikur Anbessa Specialized Hospital, Addis Ababa, Ethiopia

## Abstract

**Background:**

Job satisfaction of pharmacy professionals is appreciably related to quality of pharmaceutical care. Poor Job satisfaction is associated with low productivity, absenteeism, high turnover and reduced working hours. Little is known about job satisfaction and its related factors among pharmacy professionals in Tikur Anbessa Specialized Hospital. Therefore, the current study is aimed to assess the level of job satisfaction among pharmacy professionals working in Tikur Anbessa Specialized Hospital, Addis Ababa, Ethiopia.

**Methods:**

Institutional based cross sectional study was conducted among 80 pharmacy professionals working in Tikur Anbessa specialized hospital in Addis Ababa, Ethiopia from January to April 2019. The census sampling technique was used and data were collected using semi-structured self-administered questionnaire. Statistical analysis was carried out using the Statistical Package for the Social Sciences version 21.0.

**Result:**

Among 85 participants, 80 responded to the questionnaires completely that makes the response rate of 94%. A majority of the respondents were female (63.8%), with age group 30–39 years (57.5%), own bachelor degree (89.0%), had 1–5 years of work experience (65.0%) and provide outpatient pharmacy service (22.5%). Near to half (47.0%) of the respondents were not satisfied with their job. Only one among five of the participants feel that they are doing professional job which they enjoy and want to stay on their current working place. The least satisfaction score was obtained for staff adequacy (15.0%) and the highest satisfaction score was obtained for job relation of pharmacists with other health care professionals (74.0%).

**Conclusion:**

In the current study near to half of the hospital pharmacists were poorly satisfied on their job. High workload, inadequate salary, low respect and treat from hospital management teams, uncomfortable working environment and insufficient promotion opportunities within the hospital were mentioned as the major reasons for their poor job satisfaction. Thus, policy makers, pharmacy directors and hospital administrators, should work to reduce workload, to increase incentives and to create good working environment to improve job satisfaction and the quality of pharmaceutical care in the hospital.

## Introduction

Job satisfaction is important to an organization in terms of its positive relationship with individual performance, productivity, employee relations, physical and mental health, and life satisfaction. On the other hand, job dissatisfaction results in less productivity, high staff turnover and poor quality of service to clients [1, 2].

The health care quality can be measured through measuring the structure, process and outcome of health care system. The level of professionals’ job satisfaction is one of the quality service indicators that have been widely used to measure the process aspect. Thus, determining the level of pharmacy professionals’ job satisfaction give insight in to the quality of pharmaceutical care [3–5].

Poor job satisfaction is associated with organizational factors and poor working environment, such as inadequate staffs, high work load, poor salary, poor welfare facilities, inadequate recognition and lack of professionalism. Treatment by administrative body and social interactions, including patient contact and colleges are also related to job satisfaction [6, 7].

In developing countries’ health care system, health workers struggle to provide high-quality care to growing patient loads in increasingly challenging working conditions. Especially, in sub-Saharan Africa, pharmacists were facing shortages of supplies, poor compensation, inadequate management systems and heavy workloads [8, 9].

The hospital Pharmacy professionals in Ethiopia provide pharmaceutical services, such as clinical pharmacy services; inventory and logistics management service; dispensing services in different units, such as outpatient, inpatient, and emergency unit [10].

Ethiopia is suffering from serious pharmacy workforce scarcity; a study reporting pharmacist density of 2.38 per 100,000 populations which is the smallest number when compared to other African countries. Moreover, there is growing need for pharmacy professionals as the health care institutions continue to expand and the government looks for full implementation of clinical pharmacy service in Ethiopia [11–13]. On top of those problems, reports from different parts of Ethiopia indicated poor job satisfaction among pharmacy professionals due to inadequate compensation, inadequate management systems, heavy workloads, and lack of technical support among others [11, 13].

Hence, it is important to measure the level of pharmacy professionals’ job satisfaction to improve the quality of pharmaceutical care services. Since there is no published study on the level of pharmacy professionals job satisfaction in Tikur Anbessa specialized hospital, the results of the present study will give good opportunity for hospital management and other policy makers to take corrective action based on the information generated.

## Methods

### Study setting and period

A facility based cross-sectional study was conducted in Tikur Anbessa specialized Hospital (TASH) from January to April 2019. The Hospital is located in central Addis Ababa near to the Emigration office. TASH is the largest referral Hospital in the country and is a University teaching Hospital in both clinical and preclinical disciplines. It provides a full range of health care services including outpatient, inpatient, surgical, referral and teaching medical services that are not available in other public or private institutions. The Hospital has about 700 beds and provides health care services for 370,000–400,000 clients per year. It has 913 academic staffs, 1204 health care professionals and 900 administrative staffs. There are 85 pharmacy professionals working in the hospital.

### Study population

All health care professionals, working in Tikur Anbessa specialized Hospital, were the source population. All pharmacy professionals working in the study hospital during the study period and volunteered to fill the questionnaire were included in the study. Newly recruited pharmacy professionals with less than 6 month work experience were excluded from the study.

### Data collection tools and procedures

To assess the level of pharmacy professionals’ job satisfaction, a self-administered semi-structured questionnaire was employed. The questionnaire was adapted from previous similar studies [14, 15]. It contains two sections; socio-demographic characteristics of pharmacy professionals and the level of pharmacy professionals’ job satisfaction. The main domains included in the later section were items designed to assess satisfaction with physical working environment, inter-professional interaction and satisfaction with incentives and recognition. A five-point Likert scale, value ranging from 1 (strongly disagree) to 5 (strongly agree), was used to rate the questions. Since all except one (my formal education overqualified me for my job) of the statements were positively worded, smaller mean values was considered as lesser satisfaction. The questionnaire was prepared in simple English to make it clear and easy to understand. The questionnaire was pre-tested and all the necessary amendments were made in the structure of the instrument before the actual data collection. The collected data were checked for completeness and analyzed using Statistical Package for the Social Sciences (SPSS) Version 21. Data were described using proportion, mean and standard deviation. Respondents with an average score of less than mean value were classified as dissatisfied and those with an average score of mean value and above were considered as satisfied [16, 17].

## Result

### Socio-demographic characteristics of the participants

Among 85 participants, 80 responded to the questionnaires appropriately that makes the response rate of 94.0%. Majority of the respondents were female (63.8%), with age group 30–39 (57.5%), bachelor degree (89.0%), 1–5 years of work experience (65.0%) and provide outpatient pharmacy service (22.5%) (Table 1).

**Table 1:** Socio-demographic characteristics of pharmacy professionals working in Tikur Anbesa specialized hospital, Addis Ababa, Ethiopia, 2019 (*n* = 80)

Variables		Number (%)
Sex	Male	29 (36.2)
	Female	51 (63.8)
Age	20–29	11 (13.8)
	30–39	46 (57.5)
	> 39	23 (28.8)
Religion	Muslim	12 (15.0)
	Orthodox	49 (61.3)
	Protestant	17 (21.2)
	Other	2 (2.5)
Ethnicity	Amhara	30 (37.5)
	Oromo	25 (31.2)
	Tigre	6 (7.5)
	Other	19 (23.8)
Year of experience	1–5	52 (65)
	6–10	23 (28.8)
	11–15	1 (1.2)
	Greater than 15	4 (5.0)
Highest academic degree	Degree	71 (88.8)
	Master	9 (11.2)
Working unit	Outpatient including ARV pharmacy	20 (25.0)
	Administrative and logistics management	16 (20.0)
	Inpatient	13 (16.3)
	Oncology	10 (12.5)
	Diabetic mellitus	5 (6.2)
	Emergency	5 (6.2)
	Operation and intensive care unit	6 (7.5)
	Clinical pharmacy	3 (3.8)
	Gynecology	2 (2.5)

### Job satisfaction among pharmacy professionals

Satisfaction with working environment, professional interaction, incentives and recognition were assessed as the main domains of pharmacy professionals’ job satisfaction. When all the three domains are considered, near to half (47.0%) of the respondents were poorly satisfied with their job.

When satisfaction level within each category was consider, near to three fifth (58.0%) of the pharmacy professionals were not satisfied with working environment. Majority (83.8%) of the respondents reported that their work load is too high and 60.0% of them stated that environmental working conditions such as lighting, air condition and toilet facilities were inadequate. On the other hand almost three fourth (74.0%) of the pharmacy professionals were satisfied in job relation they have with other health care professionals (Table 2).

**Table 2:** Overall job satisfaction of pharmacy professionals working in Tikur Anbesa Specialized Hospital based on work environment, Addis Ababa, Ethiopia, 2019 (*n* = 80)

Items	Strongly disagree No. (%)	Disagree No. (%)	Neutral No. (%)	Agree No. (%)	Strongly agree No. (%)
Working environment					
The opportunity for promotion within the hospital, where I currently work is good	17 (21.2)	20 (25.0)	12 (15.0)	18 (22.5)	13 (16.3)
Employees have sufficient amount of freedom to decide how they do their work in the pharmacy	17 (21.2)	21 (26.3)	8 (10.0)	26 (32.5)	8 (10.0)
Staffing is adequate; enough employees are hired to cover the workload in the pharmacy	40 (50.0)	27 (33.8)	1 (1.2)	6 (7.5)	6 (7.5)
My supervisor has an adequate knowledge to perform his duties	28 (35.0)	8 (10.0)	23 (28.7)	13 (16.3)	8 (10.0)
There is suitable working environment (such as space, ventilation, lighting, facilities to hygiene)	27 (33.8)	21 (26.3)	9 (11.2)	20 (25.0)	3 (3.7)
The hospital management respects and treats professionals	26 (32.5)	28 (35.0)	17 (21.2)	4 (5.0)	5 (6.3)
Professional interaction					
Physicians consult me on professional Matters	13 (16.3)	23 (28.7)	14 (17.5)	25 (31.2)	5 (6.3)
Physicians cooperate when I communicate “job-related” matters with them	6 (7.5)	16 (20.0)	20 (25.0)	30 (37.5)	8 (10.0)
My fellow employees (staff working with me) treat me with professional respect	6 (7.5)	5 (6.3)	10 (12.5)	44 (55.0)	15 (18.7)
The people with whom I work are friendly	4 (5.0)	4 (5.0)	9 (11.2)	37 (46.3)	26 (32.5)
Nurses cooperate when I communicate “job-related” matters with them	6 (7.5)	6 (7.5)	13 (16.3)	46 (57.5)	9 (11.2)
Nurses often initiate consultations with me on professional matters	6 (7.5)	6 (7.5)	21 (26.3)	45 (56.2)	2 (2.5)
I am satisfied with the “on-the-job” relationships I have with others	14 (17.5)	16 (20.0)	8 (10.0)	32 (40.0)	10 (12.5)
The lay person is knowledgeable about the level of education of pharmacists	15 (18.7)	21 (26.3)	23 (28.7)	18 (22.5)	3 (3.8)
Incentive and recognition					
My salary is appropriate	44 (55.0)	21 (26.2)	4 (5.0)	6 (7.5)	5 (6.3)
My talents are fully utilized on my job	18 (22.5)	26 (32.5)	15 (18.7)	17 (21.3)	4 (5.0)
My formal education overqualified me for my job	9 (11.3)	18 (22.5)	15 (18.7)	24 (30.0)	14 (17.5)
All things considered, I am satisfied with my job	23 (28.7)	28 (35.0)	8 (10.0)	19 (23.8)	2 (2.5)
I am willing to continue the current job in future too	18 (22.5)	25 (31.3)	16 (20.0)	15 (18.7)	6 (7.5)
The time goes by quickly while I am at work	17 (21.3)	14 (17.5)	8 (10.0)	30 (37.5)	11 (13.7)
I often leave work with a feeling that I’m doing professional job which I enjoy	16 (20.0)	31 (38.7)	17 (21.3)	9 (11.3)	7 (8.7)
Knowing what I know now, if I had to decide all over again, I would still choose pharmacy	17 (21.3)	14 (17.5)	12 (15.0)	25 (31.2)	12 (15.0)
If my children were interested in pharmacy, I would encourage them to pursue it as a career	14 (17.5)	17 (21.3)	10 (12.5)	23 (28.7)	16 (20.0)

When incentive and recognition related issues are concerned, nearly to half of the pharmacy professionals (51.0%) were not satisfied. More than four in five (81.2%) of the respondents were poorly satisfied with their salary and almost half of them (47.5%) think that their formal education is overqualified them for their current job. Only one in five (20.0%) of the participants feel that they are doing professional job which they enjoy and willing to continue the current job in future too, since they feel their talents are not fully utilized (Table 2).

The main reasons mentioned for pharmacy professionals dissatisfaction were high work load (83.8%), inadequate salary (81.2%), low respect and treat from hospital management teams (67.5%), inappropriate working environment (60.0%), and insufficient promotion opportunities within the hospital (46.2%) (Table 3).

**Table 3:** Factors affecting job satisfaction of pharmacy professionals working in Tikur Anbesa Specialized Hospital, Addis Ababa, Ethiopia, 2019

Reasons for dissatisfaction	Number (%)
High work load	67 (83.8)
Inadequate salary	65 (81.2)
Low respect and treat from hospital management teams	54 (67.5)
Inappropriate working environment (such as space, ventilation, lighting, facilities and hygiene)	48 (60.0%)
Lack of freedom to decide how they do their work in the pharmacy	38 (47.5%)
Insufficient promotion opportunities	37 (46.2)

## Discussion

Job satisfaction is important to an organization in terms of its positive relationship with individual performance, productivity, employee relations, physical and mental health and life satisfaction. On the other hand, job dissatisfaction results in less productivity, high staff turnover and poor quality of service to clients [1, 2].

This study attempted to assess job satisfaction of pharmacy professionals working in Tikur Anbessa Specialized Hospital. Slightly more than half (53.0%) of the respondents were satisfied with their job (a mean score 3.1 ± 1.1) measured out of five. This finding is consistent with a study conducted on job satisfaction scale of pharmacist in Ethiopia with a mean score of 3.0 ± 1.11 [11] and a result from University of Gondar Referral Hospital (54.0%) [18].

However, the current finding is lower than the results from studies conducted on the assessment of job satisfaction among pharmacy professionals in south west Ethiopia (60.8%) and Zimbabwe, with mean score of 3.81 out of five [8, 19]. The probable reason for the difference might be the participants in south west Ethiopia and Zimbabwe includes pharmacy professionals from different settings such as private health facilities, industries and community pharmacies in addition to hospital pharmacy. The other reason might be people in Addis Ababa may expect more compared with people in south west Ethiopia, where they are better than local residents. Job satisfaction is a key factor affecting professional motivation and productivity, since satisfied workers are in better position to deliver the service to the expected standard. Consequently, it is very important for the hospital management to look deep in to the case and take appropriate measures.

In the current study, lesser satisfaction was scored for working environment and personal outlook domains than professional interaction. More than half (58.0%) of the respondents were not satisfied with working environment. This is consistent with a study conducted in eastern Ethiopia, where 52% of the pharmacy professionals were dissatisfied with the physical working environment [20]. However, this finding is lower than a study from China, where 90% of the participants were satisfied with their working environment [21]. This might be related to low socio-economic status of Ethiopia, high work load, poor hospital administrations and low payment to the pharmacist.

In the present study high workload, inadequate salary, low respect and treatment from hospital management teams, uncomfortable working environment and insufficient promotion opportunities within the hospital were mentioned as the main reasons for pharmacy professionals’ dissatisfaction. The finding of this study is comparable to the previous studies on the assessment of job satisfaction among pharmacy professionals in East Ethiopia, South-west Ethiopia and Saudi Arabia, that indicated low salary, high workload, inadequate training and education opportunities, insufficient promotion opportunities, lack of incentive, poor interaction with other health care team members and poor health institution infrastructure as the main factors for job dissatisfaction [22–24]. Hence, hospital pharmacy coordinator and hospital managers should use different techniques to decrease the work load on the pharmacist and to increase efficiency of pharmacy workforce.

Similar to research findings from Saudi Arabia, our study revealed that more than four in five (81.2%) of the pharmacists were least satisfied by the amount of payment they get for their work [23]. Salary increment was adopted as a strategy to address shortages and this combined with attractive job opportunities led to a subsequent increase in the number of students and graduates in America [25]. In support to this, a study conducted in East Ethiopia revealed that improving salary, good management and better career development as motivational factor to increase satisfaction and to decrease health professional migration [24].

Barely 26% of the study participants think that their talents are fully utilized on their job and willing to continue the current job in future too, which is an alarming that may lead to high turnover rates and withdrawal from the labor market. This is in agreement with a study conducted in Saudi Arabia, where only 34% of the hospital pharmacist considered spending the remainder of their career in their current job [23]. One possible explanation to the present finding could be the existing role of pharmacists in the hospital setting mainly focusing on product oriented function. Hence, pharmacists in hospital might perceive that they are using their skills to a lesser extent than their peers employed in the other settings. According to Kahaleh and Gaither, the ability to utilize professionals’ skill was identified as the most important factor in their perception of the ideal job [26]. In general, in an effort to enhance coverage and quality of pharmaceutical services, hospital pharmacy managers should focus on altering the job to provide greater use of skills and abilities to provide increased challenge in the work.

## Limitations of the study

This research was limited to pharmacy professionals working in Tikur Anbessa Specialized Hospital, so care should be taken while generalizing the result to all pharmacy professionals working in different sectors. In addition, the data may also be subjected to bias, since the data were collected using self-administered questionnaire.

## Conclusions

Finding from this study indicated that near to half of the hospital pharmacists were poorly satisfied on their job. High workload, inadequate salary, low respect and treat from hospital management teams, uncomfortable working environmental and insufficient Promotion opportunities within the hospital were mentioned as the major reasons for Hospital pharmacists’ dissatisfaction. Thus, policymakers, pharmacy directors and hospital administrators, should work to reduce workload, to increase incentives and to create good working environment to improve job satisfaction and the quality of pharmaceutical care in the hospital.

## Supplementary information

**Additional file 1: Annex 1.** Part I: Socio-demographic characteristic. Part II: Questions on pharmacy professionals’ level of job satisfaction. Part III: Questions related to pharmacy professionals opinion on factors related to job satisfaction.

## Data Availability

The data collection tools are attached as an additional supporting file within the text. The data sets are available from the corresponding author on reasonable request (Additional file 1: Annex 1).

## Abbreviations

TASH: Tikur Anbessa Specialized Hospital
ARV: Antiretroviral

## References

[1] Bhatnagar K, Srivastava K (2012). Job satisfaction in health-care organizations. Ind Psychiatry J.

[2] Tadese T, Mohamed A, Mengistie A (2015). Assessment of factors influencing job satisfaction among health care providers, federal police referral hospital, Addis Ababa, Ethiopia. Ethiop J Health Dev (EJHD).

[3] Donabedian A (1988). The quality of care. How can it be assessed?. JAMA.

[4] Best M, Neuhauser D, Donabedian A (2004). Father of quality assurance and poet. Qual Saf Health Care.

[5] Nau D (2009). Measuring pharmacy quality. J Am Pharm Assoc.

[6] Clegg W (1993). Psychology of employee lateness, absence and turnover. J Appl Psychol.

[7] Freeman R (1978). Job satisfaction as an economic variable. Am Econ Rev.

[8] Amy H (2009). Job satisfaction and morale in the Uganda health work force. Health Aff.

[9] Rothman S, Coetzer E (2002). The relationship between personality dimensions and job satisfaction. Manag Dyn J South Afr Inst Manag Sci.

[10] Bilal AI, Tilahun Z, Gebretekle GB, Ayalneh B, Hailemeskel B, Engidawork E (2017). Current status, challenges and the way forward for clinical pharmacy service in Ethiopian public hospitals. BMC Health Serv Res.

[11] Gebremedhin BG, Teferi GF (2013). Assessment of pharmacists workforce in Ethiopia. Ethiop J Health Dev.

[12] Henok GT, Ousam AA, Abebe BM, Akshaya SB (2018). Challenges and opportunities of clinical pharmacy services in Ethiopia: a qualitative study from healthcare practitioners’ perspective. Pharm Pract.

[13] Surur AS, Teni FS, Girmay G, Moges E, Tesfa M, Abraha M (2015). Assessment of the structural and process aspects of pharmaceutical care at a university hospital in Ethiopia. J Pharm Bioallied Sci.

[14] Eshetu E, Gedif T (2011). Quality of pharmacy services in government hospitals in Addis Ababa, Ethiopia. Ethiop Pharm J.

[15] Kerschen AM, Armstrong EP, Hillman TN (2006). Job satisfaction among staff, clinical, and integrated hospital pharmacists. J Pharm Pract.

[16] Sullivan GM, Artino AR (2013). Analyzing and interpreting data from likert-type scales. J Grad Med Educ.

[17] Norman G (2010). Likert scales, levels of measurement and the “laws” of statistics. Adv Health Sci Educ Theory Pract.

[18] Gedif G, Sisay Y, Alebel A, Belay YA (2018). Level of job satisfaction and associated factors among health care professionals working at University of Gondar Referral Hospital, Northwest Ethiopia: a cross-sectional study. BMC Res Notes.

[19] Ndlovu T, Gavaza P, Maponga C (2009). The perception of Zimbabwean pharmacists of their overall job satisfaction and the factors associated with it. East Cent Afr J Pharm Sci.

[20] Ayele Y, Hawulte B, Feto T, Basker G, Bacha Y (2020). Job satisfaction among pharmacy professionals working in public hospitals and its associated factors, eastern Ethiopia. J Pharm Policy Pract.

[21] Duan JJ, Li GC, Situ B, Tao JH (2011). Survey of career identity and job satisfaction among young hospital pharmacists in Guangdong province, China. Afr J Pharm Pharmacol.

[22] Ahmed SM, Tolera M, Angamo M (2013). Assessment of job satisfaction among pharmacy professionals in Southwest Ethiopia. Int J Pharm Sci Res.

[23] Balkhi B, Alghamdi A, Alshehri N (2017). Assessment of job satisfaction among hospital pharmacists in Saudi Arabia. J Pharm.

[24] Jundi A, Mitikie G. Assessment of the magnitude, patterns and determinant factors of health worker migration from the public health sectors: a descriptive case study in East Hararghe zone of Oromiya, Eastern Ethiopia. Addis Ababa University Digital Librar; 2012.

[25] Kenreigh CA, Wagner LT. The pharmacist shortage: where do we stand? Medscape Pharmacists 2006; 7. Available at: http://www.medscape.com. Accessed 20 June 2019.

[26] Kahaleh A, Gaither C (2007). The Effects of work setting on pharmacists' empowerment and organizational behaviors. Res Soc Admn Pharm.

