# Supplementary material for: Assessment of job satisfaction among pharmacy professionals
Source: J Pharm Policy Pract. 2021 Aug 21;14:71. doi: 10.1186/s40545-021-00356-1 (PMC8379794; doi:10.1186/s40545-021-00356-1)
Supplement: Supplementary file 1 — Additional file 1: Annex 1. Part I: Socio-demographic characteristic. Part II: Questions on pharmacy professionals’ level of job satisfaction. Part III: Questions related to pharmacy professionals opinion on factors related to job satisfaction. [file 40545_2021_356_MOESM1_ESM.docx]

**Annex 1**

**Self administered questionnaire for data collection on pharmacy professionals’ job satisfaction**

**Addis Ababa University**

**School of Pharmacy**

**Department of Pharmaceutics and social pharmacy**

**Purpose**: this study is designed to assess the level of Job Satisfaction among Pharmacy Professionals’ working in Tikur Anbessa Specialized Hospital, Addis Ababa, Ethiopia. Your answers are very important and valuable to the successful completion of this study.

**Please** be honest in filling this questionnaire, since the information you provide will be solely used for research purposes. This survey will be confidential, anonymous, and data will be analyzed in aggregates.

For comments/questions please contact me via:

Email: [muluworksahile78@gmail.com/yne50@yahoo.com](mailto:muluworksahile78@gmail.com/yne50@yahoo.com)

Mobile: +251-911-08-40-83

**Part I. Socio-demographic characteristic.**

This section contains questions that help us to understand your answers to the other sets of questions in the next section. If you are unsure about how to reply to any of the questions, please give the best answer you can and write your comments beside the question.

1. Sex

male □ female □

1. Age

20-29 □ 30-39□ above 39 □

1. Current religion

Orthodox □ Muslim □ Protestant □ Catholic □

Others □, please specify­­­­­­­­­__________________

1. Ethnicity

Oromo □ Tigre □ other□, please specify________________

1. Highest academic degree:

Diploma□ Degree □ Master□

1. Year of experience:

Less than 1 year □

1-5 years □

6-10 years □

11-15 years □

above 15 years □

1. **Working unit**

Outpatient including ARV pharmacy □

Inpatient □

Emergency □

Oncology □

Diabetic mellitus □

Operation and Intensive care unit □

Clinical pharmacy service □

Gynecology □

Administrative and logistics management□

Other □, please specify_________________

**Part II. Questions on Pharmacy Professionals’ Level of Job Satisfaction**

In this section, your feelings about your work will be asked. For each statement on the left, please encircle one number which best describes the level of your agreement (***1=Strongly Disagree (SD); 2=Disagree (D); 3=Neutral-N (Neither agree nor disagree); 4=Agree (A) and 5=Strongly Agree (SA)***)

| No. | Items | SD | D | N | A | SA |
| --- | --- | --- | --- | --- | --- | --- |
|  | The opportunity for promotion within the hospital where I currently work is good | 1 | 2 | 3 | 4 | 5 |
|  | Employees have sufficient amount of freedom to decide how they do their work in the pharmacy | 1 | 2 | 3 | 4 | 5 |
|  | Staffing is adequate; enough employees are hired to cover the workload in the pharmacy | 1 | 2 | 3 | 4 | 5 |
|  | My supervisor has an adequate knowledge to perform his duties | 1 | 2 | 3 | 4 | 5 |
|  | There is suitable working environment (like space, ventilation, lighting, facilities to hygiene) | 1 | 2 | 3 | 4 | 5 |
|  | The hospital management respects and treats pharmacy professionals | 1 | 2 | 3 | 4 | 5 |
|  | Physicians consult me on professional matters | 1 | 2 | 3 | 4 | 5 |
|  | Physicians cooperate when I communicate “job- related” matters with them | 1 | 2 | 3 | 4 | 5 |
|  | My fellow employees (staff working with me) treat me with professional respect | 1 | 2 | 3 | 4 | 5 |
|  | The people with whom I work are friendly | 1 | 2 | 3 | 4 | 5 |
|  | Nurses cooperate when I communicate “job-related” matters with them | 1 | 2 | 3 | 4 | 5 |
|  | Nurses often initiate consultations with me on professional matters | 1 | 2 | 3 | 4 | 5 |
|  | I am satisfied with the “on-the-job” relationships I have with others | 1 | 2 | 3 | 4 | 5 |
|  | The lay person is knowledgeable about the level of education of pharmacists | 1 | 2 | 3 | 4 | 5 |
|  | My salary is appropriate | 1 | 2 | 3 | 4 | 5 |
|  | My talents are fully utilized on my job | 1 | 2 | 3 | 4 | 5 |
|  | My formal education overqualified me for my job | 1 | 2 | 3 | 4 | 5 |
|  | All things considered, I am satisfied with my job | 1 | 2 | 3 | 4 | 5 |
|  | I am willing to continue the current job in future too. | 1 | 2 | 3 | 4 | 5 |
|  | The time goes by quickly while I am at work | 1 | 2 | 3 | 4 | 5 |
|  | I often leave work with a feeling that I’m doing something which I enjoy | 1 | 2 | 3 | 4 | 5 |
|  | Knowing what I know now, if I had to decide all over again, I would still choose pharmacy | 1 | 2 | 3 | 4 | 5 |
|  | If my children were interested in pharmacy, I would encourage them to pursue it as a career | 1 | 2 | 3 | 4 | 5 |

**Part III: Questions related to pharmacy professionals opinion on Factors related to Job satisfaction**

1. If you are not satisfied with your job, please specify the reason for your dissatisfaction ( more than one answer is possible)

Inadequate Salary □

High Work load □

Insufficient Promotion opportunities □

Low respect and treat from hospital management teams□

Inappropriate working environment (like space, ventilation, lighting, facilities and hygiene) □

Lack of freedom to decide how I do my work in the pharmacy □
